# Comparing botulinum toxin and biofeedback therapies for awake bruxism: a randomized clinical trial

**DOI:** 10.1186/s12903-025-07133-5

**Published:** 2025-11-11

**Authors:** Tatiana Ferreira Foscaldo, Paulo Henrique dos Santos Belo Junior, Giselle Rodrigues Ribeiro, Leilane Samary de Proença, Giancarlo De la Torre Canales, Plinio Mendes Senna

**Affiliations:** 1 Department of Prosthodontics, Rio de Janeiro State University, Rio de Janeiro, Rio de Janeiro, Brazil; 2 Department of Stomatology, Piracicaba Dental School, University of Campinas (UNICAMP), Piracicaba, São Paulo, Brazil; 3 Egas Moniz Center for Interdisciplinary Research (CiiEM), Egas Moniz School of Health & Science, Caparica, Almada, Portugal; 4 Department of Dental Medicine, Division of Oral Rehabilitation, Karolinska Institutet, 14104 Huddinge, Sweden

**Keywords:** Temporomandibular disorders, Bruxism, Myofascial pain, Ecological momentary assessment, Electromyography, Psychological biofeedback

## Abstract

**Background:**

Behavioral therapy using electromyography-based biofeedback (BIO) and botulinum toxin type A (BTA) injections are potential treatments for managing awake bruxism (AB) in patients with temporomandibular disorders (TMD). The aim of this study was to compare the effectiveness of BIO and BTA in reducing AB behaviors, pain intensity, and psychosocial distress in individuals with TMD.

**Methods:**

This single-center, single-blind, parallel-group randomized clinical trial enrolled 40 adults with TMD diagnosed with AB. AB was assessed using the Oral Behavior Checklist (OBC) and smartphone-based Ecological Momentary Assessment (EMA). Participants presenting an AB frequency of ≥ 60% were randomly allocated to receive BIO or BTA intervention. Pain intensity was measured by the Characteristic Pain Intensity (CPI) score, and psychosocial status was evaluated using the Patient Health Questionnaires (PHQ-9, PHQ-15) and the Generalized Anxiety Disorder questionnaire (GAD-7). Assessments were conducted at baseline, and at 1-, 3-, and 6-month follow-ups. Statistical analyses included Mann–Whitney U for AB behaviors, Wilcoxon Signed Rank test for within-group comparisons, and the Kruskal–Wallis test followed by the Dwass-Steel-Critchlow-Fligner post hoc test for between-group comparisons.

**Results:**

Intra-group comparisons showed a significant reduction in BIO group for sustained teeth contact (BIO: *p* = 0.004) and EMA-assessed AB global behavior (BIO: *p* = 0.008). No significant improvements were found in BTA group for the same variables in all follow-ups (*p* > 0.05). However, no significant differences were found between groups in all follow-ups for OBC and EMA scores (*p* > 0.05). Similarly, there were no significant differences between groups for CPI (*p* = 0.39) or psychosocial outcomes (*p* > 0.05).

**Conclusion:**

Even though neither intervention improved pain nor psychosocial outcomes, BIO significantly reduced AB behaviors, indicating that BIO may be preferable for controlling AB behaviors in TMD patients.

**Trial registration:**

Brazilian Registry of Clinical Trials (ReBEC), RBR-62cftbp. Registered on January 9, 2024.

## Background

Awake bruxism (AB) is an oral behavior characterized by masticatory muscle activity during wakefulness, involving repetitive or sustained tooth contact, jaw clenching, or thrusting. It is not classified as a movement disorder [1]. According to the current consensus, the focus should be on the assessment rather than the diagnosis of bruxism, given its multifactorial etiology and fluctuating nature. Assessment methods include self-reported tools (e.g., questionnaires), clinical approaches (e.g., physical examination), and device-based instruments (e.g., electromyographic recordings) [1, 2]. Specifically for AB, the use of smartphone-based Ecological Momentary Assessment (EMA) is recommended. EMA allows real-time, self-reported data collection on AB-related behaviors during periods of device usage and functions as a structured component of the assessment protocol [3–5]. In this context, the most widely used EMA tool is the BruxApp®, which prompts users to select among five standardized categories: “relaxed jaw muscles”, “sustained tooth contact” (light continuous contact without force), “clenching” (forceful tooth contact), “teeth grinding”, and “jaw bracing” (sustained masticatory muscle contraction without tooth contact). Empirical data indicate that users, after receiving appropriate instructions, can differentiate these conditions with reasonable reliability — for example, teeth contact, clenching, and bracing have been reported with distinct frequencies in studies using the BruxApp® in healthy adults [6–8]. A recent meta-analysis that applied the 2018 International Consensus criteria reported a prevalence of AB of 12.2% (95% CI: 6.32–19.64; I^2^ = 78%) [9]. Sustained tooth contact was the most frequently reported behavior (14.5%), followed by clenching (10%) [3].

Although AB is not considered a movement disorder [1], it is recognized as a potential risk factor for tooth wear, prosthetic complications, and temporomandibular disorders (TMD). Among TMD patients, the frequency of AB behaviors ranges from 50 to 62% [10–12]. EMA-based data indicate that jaw bracing is the most reported behavior (29.4%), followed by sustained tooth contact (24%), clenching (8%), and grinding (< 1%) [10], regardless of the presence of painful TMD. Individuals with AB also commonly exhibit reduced pain thresholds in the masticatory and cervical muscles, limited mandibular mobility, headaches, and impaired quality of life [13, 14]. Consequently, therapeutic strategies are recommended when AB is identified as a contributing risk factor. The first-line approach involves patient education aimed at promoting behavioral changes to reduce AB behaviors, including clarification of the mandibular rest position and encouragement to maintain it [6].

Surface electromyographic biofeedback (BIO) is an active cognitive-behavioral tool in which an electromyographic device assists patients in recognizing and maintaining mandibular relaxation through visual and/or auditory feedback based on their own muscle activity [15–17]. A recent systematic review with meta-analysis reported that BIO may reduce masticatory muscle activity related to AB in the short term. However, the available evidence is limited by a high risk of bias and a low level of certainty [18]. Additionally, only one study has investigated the effects of BIO on TMD-related pain, reporting symptom improvement based on both subjective and clinical assessments [15].

Botulinum toxin type A (BTA) has recently gained popularity in dentistry for the management of sleep bruxism due to its muscle-paralyzing effects, which reduce occlusal forces [19–21], and for TMD because of its antinociceptive properties [22–27]. While BTA may help protect orofacial structures from the potential consequences of sleep bruxism, it does not reduce the frequency of sleep bruxism episodes [19, 20]. Evidence supporting the use of BTA for AB remains scarce, despite its potential to reduce muscle force during AB behaviors. Furthermore, considering that clinical trials have reported muscle- and bone-related adverse effects associated with higher doses or repeated applications of BTA [28–33], it is essential to evaluate the efficacy and safety of low-dose protocols for AB management. Regarding the literature on the use of BTA in TMD, high-quality evidence-based data—particularly studies that take Axis II factors into consideration—remains lacking [34].

Given these considerations, identifying effective interventions to control AB behaviors and alleviate TMD-related pain is crucial. Therefore, this single-blind randomized clinical trial aimed to compare the efficacy of electromyographic biofeedback (BIO) and a low-dose BTA protocol in reducing AB behaviors and pain intensity in patients with TMD over a 6-month follow-up period. The null hypothesis was: *“There is no difference in the efficacy of BIO and BTA injection in reducing the frequency of AB behavior in patients with TMD over 6 months.*”

## Methods

### Study design and ethical aspects

This single-center, single-blind, randomized clinical trial was conducted at the Dental School of the State University of Rio de Janeiro, Brazil. The study protocol was approved by the local Research Ethics Committee (CAAE 58283722.9.0000.5259) and registered in the Brazilian Registry of Clinical Trials (ReBEC) (RBR-62cftbp, registered on 09 January 2024). The study adhered to the Declaration of Helsinki principles, and all participants provided a written and signed inform consent to participate in the trial. The reporting of data followed the Consolidated Standards of Reporting Trials (CONSORT) guidelines [35].

### Participants

Eligible participants were Brazilian adults aged 18 to 50 years, diagnosed with temporomandibular disorders (TMD) according to the Brazilian Portuguese version of the Diagnostic Criteria for TMD (DC/TMD) [36]. Awake bruxism (AB) was assessed using both non-instrumental and instrumental approaches.

The non-instrumental assessment included a detailed clinical examination to detect signs potentially associated with AB, and assessments based on the participant’s self-perception, including self-reported bruxism states, history of bruxism, and reports of related complaints (following the recommendations of the Standardized Tool for the Assessment of Bruxism [STAB] [37]). These subjective aspects of AB assessment are considered potentially valid for identifying AB in clinical and research settings [38].

The clinical examination assessed:

- Tooth wear patterns consistent with bruxism, particularly on incisal and occlusal surfaces, noting that AB typically produces localized wear from sustained tooth contact or clenching, whereas sleep bruxism more often causes generalized attrition and grinding facets.
- Masseter and temporalis muscle hypertrophy or tenderness, which may indicate frequent clenching or jaw bracing.
- Muscle pain upon palpation and reduced mandibular range of motion, more strongly associated with sustained clenching/bracing rather than light tooth contact.
- Temporomandibular joint sounds and tenderness, present in both AB and SB but not reliably differentiating the two.

The instrumental assessment involved Ecological Momentary Assessment (EMA) using the smartphone-based BruxApp® at baseline to determine AB presence and severity. The application guides participants to report their masticatory muscle activity in five categories: relaxed jaw muscles, light sustained tooth contact, forceful clenching, teeth grinding, and jaw bracing (muscle contraction without tooth contact). AB frequency was calculated based on the average occurrence of specific and global behaviors over one week, both at baseline and follow-ups.

Exclusion criteria included neurological or psychiatric disorders, recent head or neck trauma, use of medications affecting AB (e.g., selective serotonin reuptake inhibitors, antipsychotics, antidepressants, anxiolytics, muscle relaxants), as well as substance use (cocaine, methamphetamine, alcohol, tobacco) or excessive caffeine consumption. Participants without smartphones with an active data plan, necessary for the EMA, were also excluded.

The sample size was calculated using G*Power software (version 3.1.9.7; Universität Kiel, Germany). Assuming a large effect size (0.5), α = 0.05, and a power of 80%, the required sample size was 20 participants per group.

### Study protocol, randomization, and blinding

The study included five screening and intervention visits. At the first visit, participants were screened according to eligibility criteria. Eligible participants received training on the use of BruxApp®, including how to identify masticatory muscle states: (a) relaxed, (b) light tooth contact, (c) bracing/thrusting, (d) clenching, and (e) grinding. No counseling regarding bruxism management or TMD was provided at this stage to avoid influencing AB behavior.

Participants used BruxApp® for one week to record baseline AB frequency. Inclusion required at least 60% response rate to the app’s alerts (equivalent to 12 alerts/day) according to the STAB criteria [37], and an AB frequency equal to or greater than 60% [10, 39].

Eligible participants were randomly assigned to one of two intervention groups (BIO or BTA) using a computer-generated randomization list (www.randomization.com) with block sizes of four. Randomization was performed by an independent technician not involved in the study. A total of 60 patients were randomized into two distinct intervention groups: BIO Group (n = 30) and BTA Group (*n* = 30). Additionally, the administration of the interventions (Visit 2 for the BTA group; Visits 2, 3, 4, and 5 for the BIO group) was performed. Outcome assessments were conducted by an independent examiner who was blinded to group allocation throughout the study period.

**Fig. 1:**
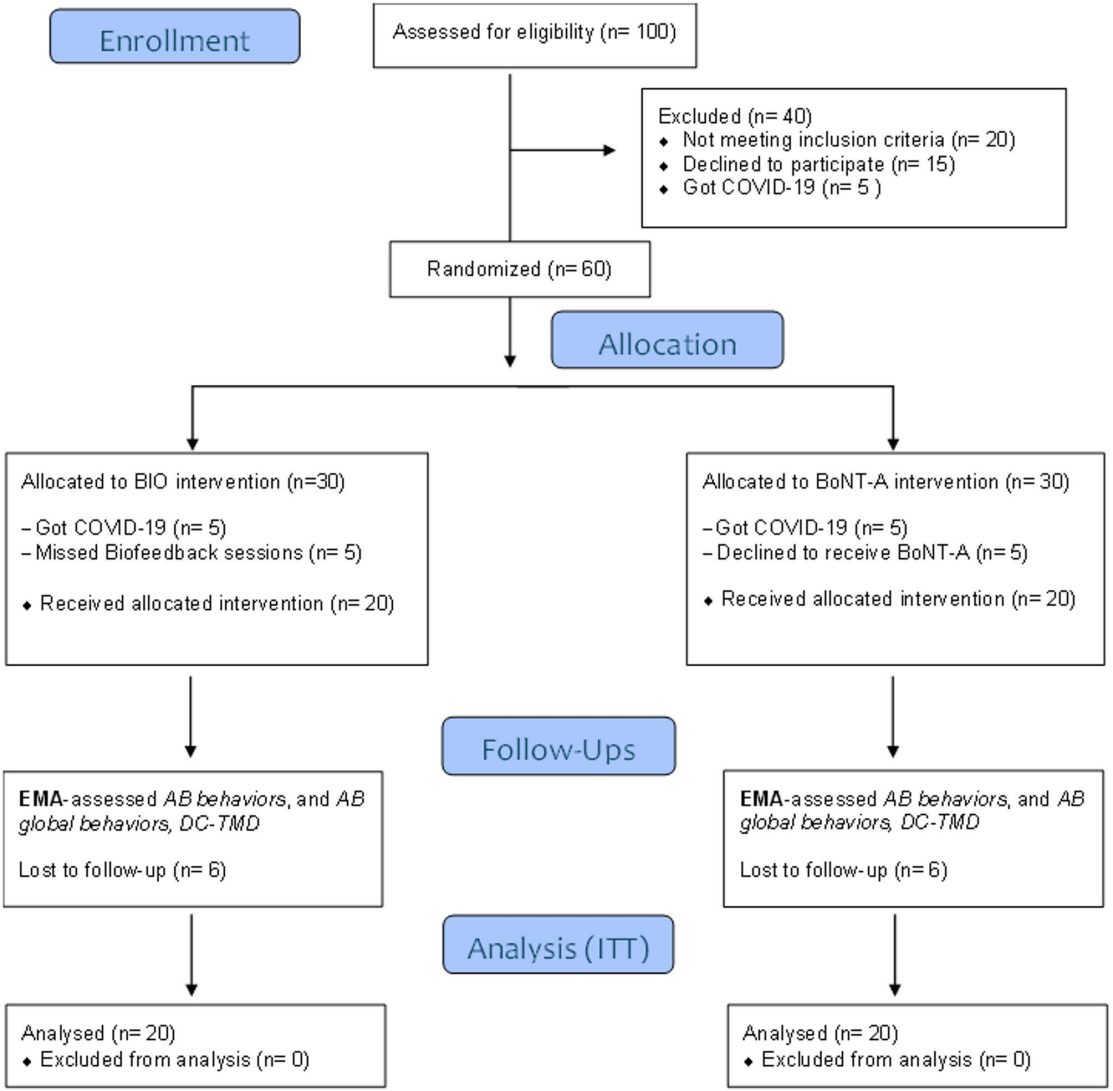
CONSORT flow diagram for patient enrollment

Follow-up assessments were conducted at 1 month (Visit 3 for BTA; Visit 6 for BIO), 3 months (Visit 4 for BTA; Visit 7 for BIO), and 6 months (Visit 5 for BTA; Visit 8 for BIO). Figure 1 presents the flowchart of patient enrollment, allocation, and follow-up. All groups underwent an additional 7-day EMA monitoring period at each follow-up time point to assess variations in specific AB behaviors measured via EMA, as well as the overall frequency of AB behaviors. After each week of data recording (baseline and follow-up), participants were instructed to generate a chart using the app interface by accessing Charts > Global Results, to print this page and send it to the researcher responsible for outcome assessments. Data regarding the frequency of AB behaviors was extracted from the printed document for statistical analysis.

### Interventions

#### Surface Electromyography Biofeedback (BIO)

BIO was performed using the Myobox® device (NeuroUp® Medical, Recife, Brazil). Participants underwent four weekly 10-min sessions conducted by a calibrated operator. Sessions were performed in the morning with participants seated, head and shoulders aligned, and the Frankfurt plane parallel to the floor, facing a screen connected to the EMG device via Bluetooth (muscle training mode > Maestro). Skin was cleaned with 70% alcohol. Bipolar surface electrodes (Ag–AgCl, 3 M Brazil) were placed over the right masseter muscle following anatomical landmarks similar to those used for BTA injections [40]. Visual and auditory feedback guided participants in relaxing their masticatory muscles through a video-game-like interface. Electromyography signals were sampled at 1,000 Hz via Bluetooth 4.0 and processed using NeuroUp® software. Filters included a 60 Hz notch filter and a Butterworth bandpass filter (30–500 Hz). Signals were processed to compute root mean square (RMS) values in real time, with peak counts normalized per minute based on individualized calibration rather than absolute EMG amplitudes.

#### Botulinum Toxin Type A (BTA)

BTA (Xeomin®, Merz Pharmaceuticals GmbH, Frankfurt, Germany) was reconstituted with 2 mL of 0.9% sterile saline to yield 5U per 0.1 mL. Each masseter received 15U divided into three injection points (5U each), and each temporalis muscle received a single injection of 5U in its anterior portion, totaling 40U per patient. Injection points for the masseter were located within an area bounded by a line from the tragus to the mouth corner, a second line 0.5 cm above the mandibular border, and two vertical lines along the anterior and posterior borders of the muscle, confirmed through functional contraction (clenching) [23]. Injections were performed by an experienced professional in facial injectable procedures not involved in any other study processes, using 1 mL syringes (BD Ultrafine®, Sigma-Aldrich) with 6 mm/30G needles for the temporalis and 13 mm/30G needles for the masseter.

### Outcomes

Patients' outcomes were assessed at four time points (baseline, 1, 3, 6 months) over the six-month study period. Both subjective assessments and objective measurements were conducted at each evaluation period by a researcher not involved in any other procedure of the study. The primary outcome was both specific and global EMA-measured frequency of AB behaviors.

#### AB behaviors: oral behavioral checklist and ecological momentary assessment

AB behavior assessment was both instrumental and non-instrumental, and performed using smartphone-based EMA and the Oral Behavior Checklist (OBC) questionnaire, respectively. The OBC consists of 21 questions regarding oral behaviors, encompassing self-reported AB behaviors over the past 30 days. For each item, participants indicate the frequency of the oral behaviors on a scale from 0 (“none of the time”) to 4 (“all of the time”). An OBC summary score of 0–16, 17–24, and 25–62 represents a normal, moderate, and severe frequency of oral behaviors, respectively [41, 42].

All groups used the BruxApp® application for EMA for 1 week at baseline and follow-ups to assess the frequency of both global and specific EMA-measured AB behaviors. Specific EMA AB behaviors were categorized into four oral conditions based on established definitions: (1) light teeth contact (continuous, low-force contact of upper and lower teeth), (2) clenching (forceful tooth contact), (3) jaw bracing (sustained masticatory muscle contraction without tooth contact), and (4) teeth grinding (rhythmic lateral or forward–backward movement of teeth).

These categories are clearly defined to allow participants to differentiate between behaviors, particularly distinguishing clenching (forceful contact) from jaw bracing (muscle contraction without tooth contact). Evidence shows that, after appropriate instruction, users can reliably report these specific behaviors using BruxApp® [38, 43, 44].

#### Pain variables

The Characteristic Pain Intensity (CPI) was assessed by numeric rating scales in the following questions (#2 to #4 from GCPS) of the “How would you rate your facial pain on a 0 to 10 scale AT THE PRESENT TIME, that is, right now”, “In the PAST SIX MONTHS, how intense was your WORST facial pain?”, and “In the PAST SIX MONTHS, on AVERAGE, how intense was your facial pain? (that is, your usual pain)”. The scales comprise a horizontal line numbered from 0, anchored by the words “no pain” at the left end, to 10, anchored by the words “worst pain imaginable” at the right end. Participants were instructed to mark the number that better represented the average pain. The CPI score was defined as the mean value of the three questions multiplied by 10 [36, 42].

The Graded Chronic Pain Scale (GCPS) v.2.0. included in the DC/TMD-Axis II was used to assess pain characteristics, interference and disability. The interference score was calculated as the mean value of questions #6 to #8 multiplied by 10. The number of disability days was recorded from question #5. Total disability points were recorded as points for disability days plus points for interference score. CPI means and total disability points were used to determine the chronic pain grade which ranges from Grade 0 to: Grade I (low intensity pain, without disability); Grade II (high intensity pain, without disability); Grade III (moderately limiting) and Grade IV (severely limiting) [36, 42].

#### Psychosocial variables

The following questionnaires were employed for data collection:

- *Patient Health Questionnaire (PHQ–9):* is used to screen symptoms of depressive mood disorders. This instrument consists of 9 questions that are each scored 0–3 according to frequency, giving a total score of 0–27. A score of 5–9 is considered as mild, 10–14 as moderate, 15–19 as moderate-severe, and 20 + as severe symptoms of depression [36, 42].
- *Patient Health Questionnaire (PHQ–15):* assesses the presence and severity of nonspecific somatic (physical) symptoms/somatization. It has 15 items that are scored on a 3-point response scale where ‘not bothered at all’ = 0 points, ‘bothered a little’ = 1 point and ‘bothered a lot’ = 2 points. Total PHQ-15 scores range from 0 to 30 points and scores of ≥ 5, ≥ 10 and ≥ 15 points specify mild, moderate, and high somatic symptoms/somatization, respectively [36, 42].
- *Generalized Anxiety Disorder – 7 (GAD-7):* is used to screen anxious mood and behavior. Its seven questions are scored exactly as the PHQ-9, giving a total score of 0 to 21. Scores of 5, 10, and 15 represent cut-points for mild, moderate, and severe anxiety, respectively [36, 42].

### Statistical analysis

Statistical analyses were performed based on the intention-to-treat principle. Data normality was evaluated using the Shapiro–Wilk test. For intra-group comparisons the Kruskal–Wallis test was applied for EMA and CPI, and ANOVA for PHQ-9, PHQ-15, GAD-7 and OBC. Regarding inter-groups comparisons, the Mann–Whitney U test was used for EMA and CPI and the Student t test for PHQ-9, PHQ-15, GAD-7 and OBC. All analyses were performed using SPSS Statistics version 20.0 (IBM Corp., Armonk, NY, USA), with the significance level set at p ≤ 0.05.

## Results

### Patient characteristics

A total of 100 individuals were initially screened for eligibility. Of these, 40 were excluded: 20 did not meet the inclusion criteria (15 due to an AB frequency below 60%, 3 due to incomplete dentition, and 2 owing to the use of exclusionary substances); 15 declined participation, and 5 were excluded due to COVID-19 infection. Among the 60 patients randomized and allocated to the study arms, 40 were included in the final analysis. Attrition occurred due to COVID-19 infection (n = 10), missed biofeedback sessions (n = 5), and refusal to receive BTA injections (n = 5). Additionally, 12 participants withdrew or failed to complete follow-up evaluations (6 from the BIO group and 6 from the BTA group) (Fig. 1).

No statistically significant differences were observed between the groups regarding demographic characteristics (Table 1). Most participants were female and self-identified as Latino. Eleven male participants were included, with 6 in the BIO group and 5 in the BTA group. The mean age was 30.3 ± 10.1 years in the BIO group and 29.6 ± 9.3 years in the BTA group. Regarding educational attainment, 45% of participants had completed higher education, and none were unemployed.

**Table 1:** Demographic and clinical characteristics of each group

	BIO	BTA	*p*-value
Participants, *n*	20	20	
Age			
Mean	30.3	29.6	0.746
Stand. deviation	10.1	9.3	
Confidence intervals 95% (lower/upper)	27.1/33.5	26.6/32.5	
Gender, *n* (%)			
Men	6 (30)	5 (25)	0.896
Women	14 (70)	15 (75)	0.832
Education, *n* (%)			
Primary school	1 (5)	2 (10)	0.687
High school	9 (45)	10 (50)	0.871
University	10 (50)	8 (40)	0.618
Annual family income, *n* (%)			
0–12.999	11 (55)	12 (60)	0.883
13.000–62.999	8 (40)	3 (15)	
63.000–92.999	1 (5)	1 (5)	
93.000–195.999	0	0	
196.000–325.999	0	3 (15)	
456.000 ou mais	0	1 (5)	
Painful TMD, *n*			
Myalgia	10	14	0.446
Miofascial pain	14	11	
Arthralgia	14	7	
Headache related to TMD	13	12	
Joint diagnoses, *n*			
Disc displacement with reduction	10	22	0.08
Disc displacement with no reduction	1	0	
Arthrosis	0	1	
Subluxation	0	0	

*TMD* Temporomandibular Disorders, *BIO* Surface electromyographic biofeedback, *BTA* Botulinum toxin type A ^*^p < *0.05*

With respect to pain-related to TMD diagnoses, 24 participants (60%) were diagnosed with myalgia, 25 (62.5%) with myofascial pain, 21 (52.5%) with arthralgia, and 25 (62.5%) reported headaches attributed to TMD. According to the DC/TMD joint diagnosis criteria, 32 participants (80%) presented with disc displacement with reduction, 1 (2.5%) with disc displacement without reduction, and 1 (2.5%) with degenerative joint disease of the temporomandibular joint.

### Oral Behavior Checklist (OBC) and Ecological Momentary Assessment (EMA)

OBC scores at baseline indicated a high frequency of maladaptive oral behaviors in both groups (Table 2). Intra-group analysis demonstrated no statistically significant reduction in OBC scores for BIO (*p* = 0.39) and BTA group (*p* = 0.32). Inter-group comparisons revealed no significant differences between groups at baseline (*p* = 0.83) or at one (*p* = 0.34), three (*p* = 0.75), and six months (*p* = 0.68) follow-ups.

**Table 2:** Oral behavior checklist (OBC) score and Awake Bruxism (AB) behaviors before and after the different treatments (mean ± standard deviation and CI)

	Follow up	BIO	BTA	*p*-value
OBC Score	Baseline	31.6 ± 9.2	30.9 ± 10.1	0.833
	CI (95%) lower/upper	27.3/35.9	26.2/35.7	
	1 month	28.40 ± 9.88	25.45 ± 9.58	0.343
	CI (95%) lower/upper	23.78/33.02	20.96/29.94	
	3 months	27.70 ± 11.15	28.75 ± 9.51	0.750
	CI (95%) lower/upper	22.48/32.92	24.30/33.20	
	6 months	26.3 ± 9.7	27.5 ± 8.9	0.686
	CI (95%) lower/upper	21.8/30.8	23.3/31.7	
	p-value	0.396	0.328	
AB Behaviors				
Bracing	Baseline	0.23 ± 0.2	0.21 ± 0.20	0.755
	CI (95%) lower/upper	0.14/0.32	0.11/0.30	
	1 month	0.16 ± 0.17	0.22 ± 0.22	0.480
	CI (95%) lower/upper	0.09/0.24	0.11/0.32	
	3 months	0.17 ± 0.19	0.21 ± 0.24	0.541
	CI (95%) lower/upper	0.08/0.26	0.10/0.32	
	6 months	0.16 ± 0.19	0.16 ± 0.20	0.849
	CI (95%) lower/upper	0.07/0.25	0.07/0.26	
	p-value	0.495	0.810	
Teeth Clenching	Baseline	0.24 ± 0.24	0.22 ± 0.17	0.957
	CI (95%) lower/upper	0.13/0.35	0.14/0.29	
	1 month	0.22 ± 0.23	0.22 ± 0.17	1.000
	CI (95%) lower/upper	0.11/0.33	0.14/0.29	
	3 months	0.22 ± 0.25	0.20 ± 0.22	0.913
	CI (95%) lower/upper	0.11/0.34	0.10/0.30	
	6 months	0.22 ± 0.24	0.25 ± 0.27	0.665
	CI (95%) lower/upper	0.11/0.34	0.13/0.38	
	p-value	0.980	0.899	
Sustained teeth contact	Baseline	0.34 ± 0.17	0.31 ± 0.16	0.735
	CI (95%) lower/upper	0.26/0.42	0.23/0.38	
	1 month	0.24 ± 0.16	0.26 ± 0.13	0.3364
	CI (95%) lower/upper	0.16/0.32	0.20/0.32	
	3 months	0.17 ± 0.11	0.27 ± 0.18	0.098
	CI (95%) lower/upper	0.12/0.23	0.19/0.36	
	6 months	0.17 ± 0.14	0.23 ± 0.14	0.099
	CI (95%) lower/upper	0.10/0.24	0.16/0.30	
	p-value	0.0045^*^	0.528	
Teeth Grinding	Baseline	0.00 ± 0.01	0.03 ± 0.09	0.005^*^
	CI (95%) lower/upper	0.00/0.02	−0.01/0.07	
	1 month	0.01 ± 0.02	0.02 ± 0.04	0.364
	CI (95%) lower/upper	0.00/0.01	0.00/0.04	
	3 months	0.01 ± 0.01	0.01 ± 0.03	0.945
	CI (95%) lower/upper	0.00/0.01	0.00/0.02	
	6 months	0.01 ± 0.01	0.03 ± 0.06	0.701
	CI (95%) lower/upper	0.00/0.01	0.00/0.05	
	p-value	0.203	0.623	
AB Global behavior	Baseline	0.81 ± 0.15	0.76 ± 0.13	0.193
	CI (95%) lower/upper	0.74/0.89	0.70/0.82	
	1 month	0.63 ± 0.27	0.69 ± 0.14	0.860
	CI (95%) lower/upper	0.50/0.76	0.63/0.76	
	3 months	0.58 ± 0.29	0.69 ± 0.18	0.364
	CI (95%) lower/upper	0.44/0.71	0.61/0.78	
	6 months	0.56 ± 0.28	0.67 ± 0.19	0.218
	CI (95%) lower/upper	0.43/0.69	0.58/0.76	
	p-value	0.008^*^	0.277	

*OBC* Oral behavior checklist, *AB* Awake Bruxism, *BIO* Surface electromyographic biofeedback, *BTA* Botulinum toxin type A, *CI* Confidence intervals

^*^p < 0.05

Additionally, EMA data demonstrated significant intra-group reductions for sustained teeth contact behavior (*p* = 0.004) and AB global behavior (*p* = 0.008) only for BIO group. Inter-group comparisons at baseline revealed significantly higher scores for teeth grinding behavior in the BTA group compared to the BIO group (*p* = 0.005), but no differences in global AB behavior (*p* = 0.73). At all follow-ups, no significant differences were observed between groups for either specific (*p* > 0.05) or AB global behaviors (*p* = 0.09) (Table 2).

### Pain variables

CPI scores did not show significant intra-group differences between baseline and all follow-ups for either BIO (*p* = 0.83) or BTA (*p* = 0.75) groups. Similarly, no significant differences were found in the inter-group analysis in all assessed time points (*p* > 0.05) (Table 3).

**Table 3:** Pain intensity (CPI score) before and after treatments (mean ± sd and CI) and number of patients and percentage (%) regarding different ratings of pain-related disability (GCPS) before and after treatments

	Follow up	BIO	BTA	p-value
**CPI**	Baseline	37.3 ± 27.5	33.0 ± 28.71	0.625
	CI (95%) lower/upper	24.5/50.2	19.56/46.44	
	1 month	31.17 ± 24.43	23.67 ± 20.32	0.566
	CI (95%) lower/upper	19.73 ± 42.60	14.16/33.17	
	3 months	31.17 ± 26.74	28.33/23.23	0.880
	CI (95%) lower/upper	18.65/43.68	17.46/39.21	
	6 months	37.0 ± 28.0	28.33 ± 24.05	0.391
	CI (95%) lower/upper	23.90/50.10	17.08/39.59	
	*p*-value	0.839	0.755	
Grade				
0/None	Baseline/ 6 months	2 (3)	5 (5)	0.638
I/low-intensity pain	Baseline/6 months	9 (11)	7 (10)	
II/High Intensity pain, no disability	Baseline/ 6 months	3 (2)	2 (2)	
III/Moderately Limiting	Baseline/ 6 months	5 (3)	5 (3)	
IV/Severely Limitating	Baseline/ 6 months	1 (1)	1(1)	

*CPI* Characteristic Pain Intensity, *BIO* Surface electromyographic biofeedback, *BTA* Botulinum toxin type A, *CI* Confidence intervals

^*^p < 0.05

Regarding GCPS scores, most participants were categorized as Grade I (40%) or Grade III (25%). No statistically significant intra- or inter-group differences were identified across the study period (p > 0.05).

### Psychosocial variables

Baseline assessments of psychosocial parameters revealed mild to moderate levels of anxiety, depression, and somatization in both groups. Intra- and inter-group analyses indicated no statistically significant changes in GAD-7, PHQ-9, or PHQ-15 scores across the study period (*p* > 0.05) (Table 4).

**Table 4:** Psychosocial findings (Axis II) across the assessments for each group

	Follow up	BIO	BTA	*p*-value
PHQ-9	Baseline	10.89 ± 6.07	13.06 ± 5.33	0.263
	CI (95%) lower/upper	7.87/13.91	10.41/15.71	
	1 month	9.78 ± 6.07	10.50 ± 4.00	0.676
	CI (95%) lower/upper	6.76/12.80	8.51/12.49	
	3 months	8.65 ± 5.27	11.50 ± 5.79	0.138
	CI (95%) lower/upper	5.94/11.36	8.62/14.38	
	6 months	10.39 ± 6.71	11.33 ± 5.75	0.653
	CI (95%) lower/upper	7.05/13.73	8.47/14.19	
	*p*-value	0.724	0.533	
PHQ-15	Baseline	9.7 ± 5.11	9.90 ± 4.87	0.900
	CI (95%) lower/upper	7.31/12.09	7.62/12.18	
	1 month	9.70 ± 5.69	9.35 ± 4.89	0.836
	CI (95%) lower/upper	7.04/12.36	7.06/11.64	
	3 months	9.45 ± 4.85	9.95 ± 5.15	0.753
	CI (95%) lower/upper	7.18/11.72	7.54/12.36	
	6 months	9.15 ± 4.79	9.60 ± 5.57	0.786
	CI (95%) lower/upper	6.91/11.39	6.99/12.21	
	*p*-value	0.984	0.980	
GAD-7	Baseline	9.44 ± 5.51	12.37 ± 5.18	0.105
	CI (95%) lower/upper	6.70/12.19	9.87/14.86	
	1 month	9.11 ± 5.40	10.26 ± 4.27	0.468
	CI (95%) lower/upper	6.50/11.71	8.21/12.32	
	3 months	8.32 ± 5.08	10.47 ± 4.74	0.184
	CI (95%) lower/upper	5.87/10.76	8.19/12.76	
	6 months	8.21 ± 5.51	10.37 ± 4.97	0.213
	CI (95%) lower/upper	5.55/10.87	7.97/12.76	
	*p*-value	0.873	0.481	

Data are presented in mean ± standard deviation and CI: Confidence intervals

*BIO* Surface electromyographic biofeedback, *BTA* Botulinum toxin type A; PHQ-9, PHQ-15: Patient Health Questionnaires; GAD-7: Generalized Anxiety Disorder questionnaire; CI: Confidence intervals

^*^p < 0.05

## Discussion

The primary finding of this study is that BIO group diminished sustained teeth contact and AB global behavior. However, throughout the study, there were no significant differences between groups across AB variables. Moreover, neither intervention yielded improvements in pain intensity or psychosocial functioning, with no differences between groups. Our results suggest that BIO treatment has greater impact on behavioral conditions related to AB, whereas the effects on pain and broader psychosocial domains appear limited for both treatments.

In the current investigation, only BIO reduced sustained teeth contact, and EMA-assessed AB global behavior after six months. However, no major differences were observed between groups over time. Previous studies have reported reductions in oral behaviors with behavioral interventions such as EMI [2, 16, 45] and EMG biofeedback [16, 17], primarily by enhancing individuals’ awareness of non-functional activity and promoting behavioral change through self-regulation and muscle relaxation. However, the evidence base for BIO therapy remains relatively limited, largely due to the lack of standardized protocols [46]. Despite this limitation, literature highlights biofeedback’s potential to produce faster and more substantial reductions in muscle activity [16, 17]. The specific features of the BIO intervention—centered on behavioral modification through increased self-awareness—may account for the intra-group improvements observed in sustained tooth contact, and global EMA behavior in our study.

Among the distinct types of AB, EMA data revealed improvements exclusively in the BIO group, specifically in sustained teeth contact, identified as the most prevalent oral behavior in healthy adults [3] and the second most common in individuals with TMD [10]. In this study, it was also the most frequent behavior at baseline, which may explain why it was the only specific behavior to significantly decrease in BIO group. This finding suggests that sustained contact may be more amenable to modification—or more easily detected and voluntarily controlled by patients—compared to behaviors such as clenching or grinding, which are typically more automatic and less consciously perceived. These results support the notion that AB should not be considered a homogeneous construct. Future research should adopt multimodal assessment strategies, such as combining EMA with surface electromyography (EMG), to better capture the nuances of each behavior and to evaluate whether certain modalities are more responsive to behavioral or pharmacological interventions.

To our knowledge, this is the first study to evaluate the effects of BTA injections on AB behaviors, which limits direct comparisons with existing literature. Prior research has largely focused on sleep bruxism, including studies using polysomnography, which have not shown significant reductions in bruxism parameters following BTA administration—only reductions in the intensity of voluntary clenching have been noted [19, 20]. Furthermore, in the present study, the degree of muscle contraction reduction achieved with the administered BTA dose was insufficient to reduce AB behaviors. This suggests that while BTA can inhibit peripheral muscle activity, it may not adequately address the central mechanisms contributing to AB. In contrast, BIO may foster increased sensorimotor awareness and conscious regulation of masticatory muscle activity. Moreover, by engaging cortical circuits involved in attention and motor inhibition, BIO may help disrupt maladaptive muscle activity and promote behavioral self-regulation [15, 16]. Thus, the different mechanisms of action—peripheral inhibition via BTA versus behavioral modulation via BIO—could explain the outcomes observed. Future studies should explore whether combining these treatments may produce synergistic effects, particularly in individuals with high-frequency AB behaviors.

It could be hypothesized that BTA would demonstrate superior effects at 1 and 3-month follow-ups, given its cholinergic mechanism of action at muscle fiber level, which is expected to yield greater efficacy at these time points. However, the 1- and 3-month results revealed no significant impact on any of the variables analyzed in the BTA group. This finding suggests that, despite its neuromuscular effects, BTA may not substantially influence the behavioral components of AB targeted in this study. It is worth noting that the BTA results in this study may have been influenced by the low dosage employed. However, there is growing evidence of potential adverse effects of BTA on masticatory muscles, including muscle atrophy [30, 31] reduced cortical thickness and bone volume [22, 32, 33], and decreased masticatory performance [22], despite its neuromuscular and analgesic properties. These effects appear more pronounced at doses exceeding 30 U per masseter and 10 U per temporalis, or with repeated applications [22, 28, 29]. In the present study, a total of 40 U per patient was used (15 U per masseter and 5 U per anterior temporalis), which is lower than commonly used doses [47, 48]. This conservative approach aimed to ensure safety by minimizing the mentioned dose-dependent adverse effects [49]. Although no reduction in masticatory performance was observed after six months (assessements performed with multiple-sized method—data not shown), it is important to emphasize that only a single injection was administered. The absence of standardized protocols and considerable variability in doses complicate the identification of safe and effective protocols for bruxism and TMD [21, 34]. Future research should evaluate the safety and efficacy of both higher and repeated low dose of BTA injections for these conditions.

There has been considerable misunderstanding in the literature regarding the primary purpose of using BTA in patients with TMD and bruxism—namely, whether the aim is to reduce bruxism activity itself or to manage the consequences of this activity, such as TMD-related symptoms. Early studies in this field often lacked well-defined inclusion criteria, which contributed to this ambiguity. The main objective of our trial was to evaluate whether BTA could influence AB behavior directly, while TMD-related symptoms were assessed as secondary outcomes. Previous research on BTA for TMD has predominantly focused on myogenous conditions, largely due to its muscle-relaxing and antinociceptive properties. Nevertheless, we considered it important to also investigate whether a potential reduction in AB could have an impact on all sub-types of TMDs, thereby broadening the scope of clinical relevance [50, 51].

Although a more pronounced reduction in pain was anticipated in the BTA group due to its known analgesic effects—independent of its neuromuscular mechanism [22–27]—no impact on CPI parameters was observed in either group. This finding contrasts with prior studies demonstrating pain reduction in patients with myofascial TMD receiving low-dose BTA injections [22, 26, 27]. However, this discrepancy may stem from the characteristics of our study sample, which included various painful TMD diagnoses rather than exclusively myofascial TMD pain. Given that BTA was administered to the masticatory muscles, the high prevalence of arthralgia (52.5%) may have attenuated its effects. Additionally, the dosage used may have been insufficient for achieving significant analgesia. Previous studies reporting pain reductions have used doses ranging from 20–25 U per masseter and 10–15 U per temporalis for sleep bruxism and myofascial pain [22, 52] and up to 75 U per masseter and 20 U per temporalis in myofascial cases [22, 23].

Muscle contractions associated with AB may be linked to stress and anxiety regulation via complex central nervous system interactions involving the limbic system, prefrontal cortex (PFC), and motor regions [53, 54]. Several studies have documented associations between AB and elevated stress, depression, anxiety [55–58], and reduced oral health-related quality of life [55]. In line with these findings, our study sample presented moderate levels of anxiety, depression, and non-specific physical symptoms. These psychological factors may be associated with the high prevalence of AB and TMD pain in our population [55–58]. However, neither treatment influenced psychosocial parameters over the course of the study. This may be explained by the absence of interventions specifically targeting psychosocial factors. Future studies should consider integrating behavioral therapies with stress and anxiety management strategies to address AB behaviors, TMD pain, and psychosocial comorbidities in a more comprehensive manner.

In addition to treatment outcomes, certain sample characteristics merit attention. OBC and EMA baseline data revealed a high frequency of non-functional oral behaviors, likely influenced by the inclusion criterion of ≥ 60% AB frequency. This finding is clinically relevant, as OBC scores above 25 are 17 times more prevalent in TMD patients and constitute a significant risk factor for TMD onset [41]. Both groups exhibited baseline AB frequencies of approximately 78.5%, markedly higher than the 62.1% reported in prior TMD studies [10]. Sustained tooth contact (32.5%) was the most common behavior, followed by clenching (23%) and bracing (22%), mirroring findings in individuals with high anxiety and TMD [10, 56]. Sustained contact and bracing are well-documented as highly prevalent in TMD populations [10, 11]. These features suggest a possible ceiling effect, particularly for participants with already elevated AB frequencies at baseline, potentially limiting the capacity to detect further improvements. As such, while no differential effectiveness was observed in this study, future investigations involving participants with lower baseline AB frequencies may yield different results. In addition, although the DC/TMD criteria provide important standardization for research, it is limited in representing the complexity of patients who usually present with multiple overlapping symptoms. Broader frameworks, such as the International Classification of Orofacial Pain (ICOP), allow for a more comprehensive view of TMDs and related conditions. Incorporating such approaches may improve communication between research findings and real-life clinical practice [59].

Finally, the relationship between oral behaviors and TMD remains complex and multifactorial, far beyond a simple causal link, and modulated by biopsychosocial factors, comorbidities, and individual risk profiles [40, 60, 61] Our sample exhibited a high prevalence of painful TMD affecting both muscle and joint structures, with no difference across TMD subtypes. This is consistent with prior literature indicating musculoskeletal pain as the most common symptom among AB individuals [61, 62]. We also found a high prevalence of disc displacement with reduction, a condition accounting for approximately 41% of clinical TMD diagnoses [63] and commonly reported among TMD patients [61, 63]. Previous studies have shown that patients with painful joint clicking exhibit higher AB scores compared to those with painless clicking [61, 63]. In alignment, our population presented high OBC and EMA AB scores alongside elevated rates of disc displacement with reduction (80%) and arthralgia (52.5%).

Only BIO group revealed some improvement in AB activity in our painful TMD sample, therefore the adoption of more conservative approaches—less complex, less expensive, and free of adverse effects—is strongly advised. The recent INfORM/IADR [64] key points for good clinical practice emphasize patient-centered decision-making and the importance of evidence-based, reversible interventions in the management of TMDs. Overtreatment, often resulting from exaggerated diagnoses and reliance on unvalidated concepts, can lead to unnecessary, invasive, and costly interventions. Such approaches expose patients to significant financial, biological, and psychosocial burdens, including greater expenses, longer treatment durations, discomfort, technical complications, and sometimes irreversible harm [65]. Moreover, they convey a misleading message that all pain problems must be "cured" by the clinician, rather than managed collaboratively with modern, conservative pain approaches. Therefore, ethical responsibility requires clinicians to prioritize conservative, evidence-based, reversible care tailored to the patient's diagnosis and needs [64–66].

### Study limitations and strengths

Several limitations must be acknowledged. First, the study included only TMD patients with high AB frequency (commonly seen in clinical practice), which may have influenced the magnitude of improvement observed. Second, the exclusion of participants using antidepressants and those without access to mobile devices may limit generalizability. Additionally, the BTA dosage used was lower than that in most prior studies assessing its efficacy for sleep bruxism and TMD-related pain. Future studies should explore the effects of higher BTA doses in patients with AB behaviors and TMD, while also monitoring potential adverse effects. A key strength of this study is the use of validated tools for AB assessment. Furthermore, to our knowledge, this is the first study to compare two distinct AB therapies over a six-month follow-up period, making it a pioneering contribution to AB literature, however longer assessment periods are recommended for future clinical trials [67–69].

## Conclusion

Within the limitations of this randomized clinical trial, BIO reduced AB behaviors, whereas both treatmentd did not improve pain intensity or psychosocial impairment, reinforcing that TMD pain requires multifactorial management beyond AB-focused interventions. Additionally, the lack of superiority of BTA, coupled with its potential adverse effects and the behavioral etiology of AB, supports the preferential use of conservative, self-regulatory approaches such as biofeedback in clinical practice.

## Data Availability

All data are available upon reasonable request to the corresponding author.
